# Effects of Natural *Rheum tanguticum* on the Cell Wall Integrity of Resistant Phytopathogenic *Pectobacterium carotovorum* subsp. *Carotovorum*

**DOI:** 10.3390/molecules27165291

**Published:** 2022-08-19

**Authors:** Yanjiao Qi, Mingyang Wang, Bo Zhang, Yue Liu, Jiaqin Fan, Zifan Wang, Li Song, Peer Mohamed Abdul, Hong Zhang

**Affiliations:** 1 Key Laboratory for Utility of Environment-Friendly Composite Materials and Biomass, Universities of Gansu Province, Lanzhou 730000, China; 2 China-Malaysia National Joint Laboratory, Biomedical Research Center, Northwest MinZu University, Lanzhou 730000, China; 3 Gansu Provincial Biomass Function Composites Engineering Research Center, Lanzhou 730000, China; 4 College of Plant Protection, Nanjing Agricultural University, Nanjing 210095, China; 5 Gansu Hualing Dairy Co., Ltd., Ganna 747000, China; 6 Department of Chemical and Process Engineering, Faculty of Engineering and Built Environment, Universiti Kebangsaan Malaysia, Bangi 43600, Selangor, Malaysia

**Keywords:** *Pectobacterium carotovorum*, *Rheum tanguticum*, resistant, morphology, antibacterial effect, virulence

## Abstract

The abuse of agricultural antibiotics has led to the emergence of drug-resistant phytopathogens. Rifampicin and streptomycin and streptomycin resistance *Pectobacterium carotovorum* subsp. *carotovorum* (*Pcc*S1) was obtained from pathological plants in a previous experiment. *Rheum tanguticum*, derived from the Chinese plateau area, exhibits excellent antibacterial activity against *Pcc*S1, yet the action mode has not been fully understood. In present text, the cell wall integrity of the *Pcc*S1 was tested by the variation of the cellular proteins, SDS polyacrylamide gel electrophoresis (SDS-PAGE), scanning electron microscopy (SEM) and Fourier transform infrared spectrophotometer (FTIR) characteristics. Label-free quantitative proteomics was further used to identify the DEPs in the pathogen response to treatment with *Rheum tanguticum* Maxim. ex Balf. extract (abbreviated as RTMBE). Based on the bioinformatics analysis of these different expressed proteins (DEPs), RTMBE mainly inhibited some key protein expressions of beta-Lactam resistance, a two-component system and phosphotransferase system. Most of these membrane proteins were extraordinarily suppressed, which was also consistent with the morphological tests. In addition, from the downregulated flagellar motility related proteins, it was also speculated that RTMBE played an essential antibacterial role by affecting the swimming motility of the cells. The results indicated that *Rheum tanguticum* can be used to attenuate the virulence of the drug-resistant phytopathogenic bacteria.

## 1. Introduction

Phytopathogenic *Pectobacterium carotovorum* subsp. *carotovorum* (*Pcc*, formerly called *Erwinia carotovora* subsp. *carotovora*) is a Gram-negative bacterium that can cause wilt, soft rot and blackleg on several plants, such as celery, potato, broccoli, carrots, etc. [1,2]. As one of the top 10 plant pathogenic bacteria based on scientific/economic importance, this pathogen causes a serious loss of product quality during its growth, transit and even storage [3]. Synthetic pesticides and agricultural antibiotics are always used to protect crops against these bacteria. However, it was indicated that the excessive utilization of pesticide can not only lead to human cancers, leukemia and asthma [4,5,6] but also pose a great threat to the emergence of resistant bacteria [7]. Nowadays, some toxic chemical pesticides are restricted, and environmentally friendly natural pesticides are urgently encouraged.

The main pathogenicity determinant of *Pcc* was plant cell wall-degrading enzymes (PCWDEs), involving pectate lyase (Pel), polygalacturonase (Peh), cellulase (Cel) and protease (Prt) [8,9]. It has been previously reported that ClpP, FlgK and MreB played essential roles in the virulence of plant pathogens. The *Pectobacterium carotovorum* subsp. *carotovorum* strain (*Pcc*S1) was obtained from pathological summer-flowering calla lily (*Zantedeschia* spp.) and induced rifampicin and streptomycin resistance in our previous experiment [10]. With the availability of genomic sequences for this rifampicin and streptomycin-resistant *Pcc*S1, the virulence determinants and the protein expression patterns in the host plant were also investigated [11]. However, the change of protein expression patterns on *Pcc*S1 influenced by inhibitors, even multi-drugs, remains poorly understood.

Natural medicine extracts are attractive alternatives to synthetic pesticides due to their low residue and environmental degradability. For example, *A. saligna* flower extract, *L. camara* leaf extract, *E. camaldulensis* bark ButE and *C. viminalis* flower ButE can inhibit the growth of phytopathogenic bacteria *P. carotovorum* compared with the positive control tobramycin [12,13,14]. As one of the three genuine rhubarbs in Chinese pharmacopoeia, *Rheum tanguticum* Maxim. ex Balf. contains several chemical components, such as anthraquinones, anthrones, saccharides, stilbenes and tannins. Rhein, emodin, aloe-emodin, physcion and chrysophanol are well-recognized as biologically active compounds [15]. Rhubarb was traditionally used as a folk medicine in China and was confirmed to have strong antibacterial, antipyretic, antiaging, antipasmolytic and cardiovascular protection effects [16,17,18]. However, to the extent of our knowledge, there are no reports in the literature regarding the identification of the antibacterial effect of rhubarb against *Pcc*. Based on our previous drug sensitivity screening experiments from more than 30 herbs, the *Rheum tanguticum* Maxim. ex Balf. extract (abbreviated as RTMBE) was first detected to remain the best antibacterial activity against this resistant *Pcc*S1 and was thus selected in the present study to obtain a detailed inhibitory effect by SDS-PAGE, SEM and FTIR analysis. In addition, the label-free based LC-MS/MS quantitative proteomic approach was also adopted to analyze the global protein alteration of the *Pcc*S1 in response to RTMBE treatment. This may help us develop some potential lead compounds against antibiotic-resistant bacterial infection and discover new pesticide targets.

## 2. Results and Discussion

### 2.1. UPLC-ESI-MS Analysis of RTMB Active Ingredients

**Figure 1:**
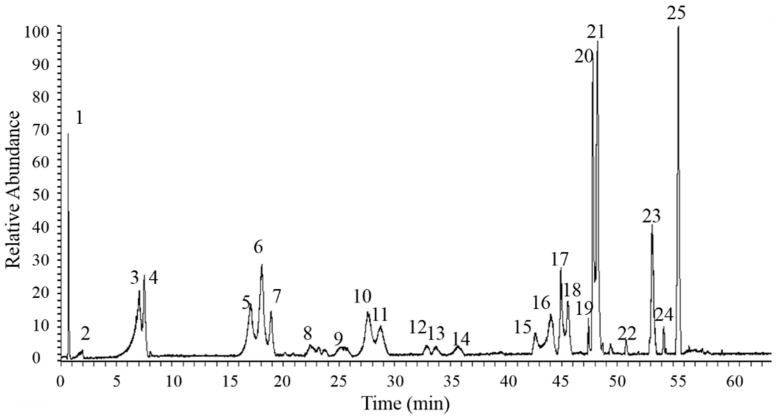
Base peak chromatograms of RTMBE.

UPLC was performed on a Thermo Scientific (Waltham, MA, USA) reversed-phase according to the chromatographic conditions described in Section 2.2. Due to more information that can be observed in the negative ion mode, it was chosen for the MS analysis rather than the positive ion mode. Finally, the components of the total 25 peaks in the total ion current (TIC) chromatogram were identified (Figure 1). The baseline of the RTMBE sample was stable, and the chromatographic peaks of each active ingredient were separated perfectly. Based on a comparative analysis of the retention time, *m*/*z* of [M-H]^−^ and MS/MS fragmentation patterns with the reference substances [15,19,20,21,22,23,24], the major bioactive constituents can be classified into six categories (Table 1), including tannins (**1**–**4** and **7**); butyrophenones (**5**, **6**, **17**, **18**, **20** and **21**); naphthoside (**8**); stilbenes (**10** and **11**); naphthopyrones (**12**) and the most abundant anthraquinone compounds (**9**, **13**–**16**, **19** and **22**–**25**). The analysis indicated that the anthraquinones compounds are the main antibacterial active ingredients [25,26]. In addition, it was also suggested that emodin and aloe-emodin derivatives could be considered as promising lead compounds for further investigations as anticancer drugs [27,28].

### 2.2. The MIC and the Growth Curves of PccS1

**Figure 2:**
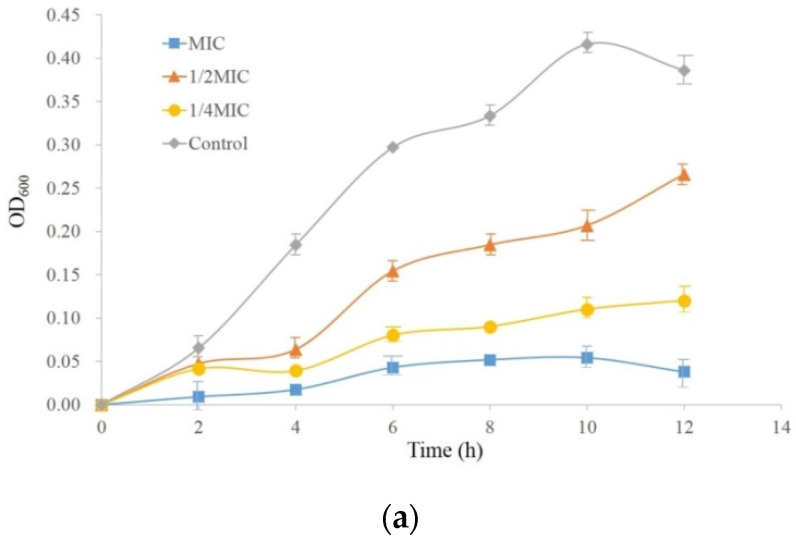

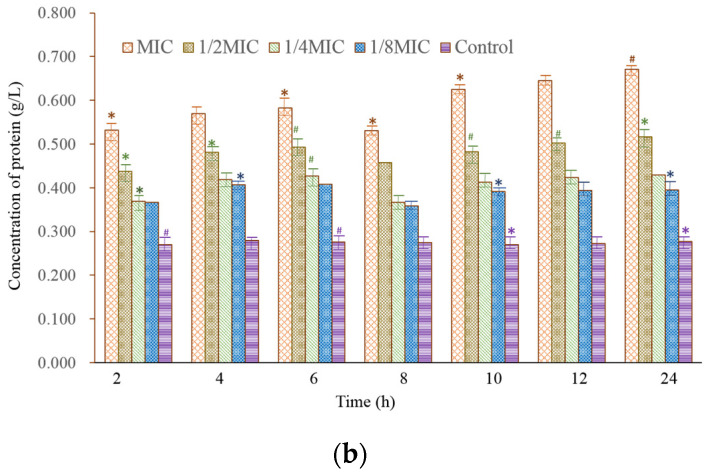
Effects of RTMBE on the bacterial growth (**a**) and extracellular protein concentration (**b**). Cells treated with the same volume of PBS were used as the control. Values are the mean ± SEM of three repeats and significantly different from the control group (* *p* < 0.05 and ^#^ *p* < 0.01).

Using the twofold dilution method, the analysis showed that the MIC value of the RTMBE was 24.96 mg/mL, whereas the solvent dimethylsulfoxide (DMSO) had no effect on the growth of *Pcc*S1. As shown in the growth curves Figure 2a, *Pcc*S1 in the control group entered the rapid growth logarithmic phase after 2 h, and the growth rate slowed down gradually after 10 h. The 1/4 × MIC and 1/2 × MIC RTMBE could partially delay the growth of the *Pcc*S1, especially during the first 4 h, while the bacterial concentration thereafter increased rapidly. It was worth noting that the growth was completely inhibited at 1 × MIC during a relatively long incubation period of 12 h. To observe the changes of the intracellular proteins, the 1/4 × MIC after incubating for 8 h was chosen for a further proteomics assay in this study based on the minimal effective concentration principle.

The extracellular protein content was also examined as a degree of cell injury and nonselective micropore formation [29]. Compared with the untreated group, the extracellular protein concentrations of other groups were changed apparently. The protein concentration increased with the increment of the drug concentration at the first 6 h, and the highest protein concentration was 0.583 g/L when treated with 1 × MIC RTMBE. This suggested that RTMBE changed the intracellular membrane permeability and caused the release of the intracellular protein. Then, the protein concentrations were gradually decreased and reached the lowest values at 8 h, which suggested that a small part of the protein was consumed by the bacteria during the growth process. This may be ascribed to the bacterial cell self-repairing functions. The protein concentrations were gradually increased again after 8 h, and the maximum value of 0.671 g/L was obtained after 24 h when the sample was treated with 1 × MIC RTMBE, which indicated that the bacterial cell structure was probably destructed and the intracellular substances consequently flowed out.

### 2.3. Fourier Transform Infrared Spectrophotometer (FTIR) Analysis

**Figure 3:**
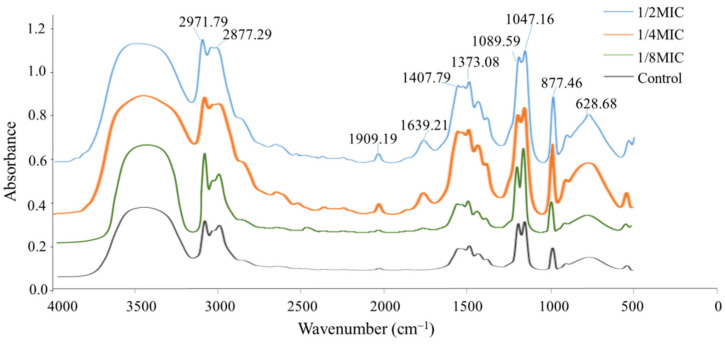
FTIR spectra of *Pcc*S1 treated by RTMBE. Cells treated with the same volume of phosphate-buffered saline were used as the control.

FTIR is considered as a comprehensive and sensitive method to detect the molecular changes in cells [30]. From Figure 3, it is shown that these FTIR spectra were generally similar, whereas the most differences were observed particularly in the complex spectral region at 1700–700 cm^−1^ and 3100–2200 cm^−1^. A noticeable variation of the bands at 2971.79 cm^−1^ was attributed to the asymmetric CH_2_ stretching of lipids, and the band around 2877 cm^−1^ was identified as the symmetric CH_3_ stretching of lipids [30]. For both the control and treated groups, the absorbance of dominant bands at 1639.21 cm^−1^ was attributed to protein amide I bands, which were gradually increased along with the increased concentrations. The bands at 1407.79 cm^−1^ and 1373.08 cm^−1^ were attributed to the asymmetric and symmetric CH_3_ bending modes of the end ethyl groups and branched methyl groups of the proteins and lipids, respectively [31], which showed a significant enhancement in the intensity of all the treated samples. The bands at 1089.59 cm^−1^ were attributed to stretching O-H coupled with bending the C-O of the polysaccharides capsule and peptidoglycan [31].

The absorption peak at 1909.19 cm^−1^, ascribed to carbonyl groups, was gradually increased with the increased RTMBE concentration. The band was also significantly increased at 1047.16 cm^−1^, which can be ascribed to the vibrational modes of -CH_2_OH and the C-O stretching vibration coupled to the C-O bending mode of cell carbohydrates [32]. The spectral region between 1300 and 900 cm^−1^ was characterized by vibrational features of cellular proteins, nucleic acids, cell membranes and cell wall components [31]. With the increase of the drug concentration, the integral strain structure was gradually collapsed, which might be characterized by stretching vibrations of polysaccharides in the cell wall peptidoglycan layer and lipopolysaccharide outer leaflet. It was also observed that the absorbance was decreased after incubating with RTMBE in the narrow region centered at 628.68 cm^−1^, which represents N-H bending of proteins [31]. The primary function of the bacterial cell wall is known to maintain the inherent shape of the bacteria and protect itself against the hypotonic environment. Once the original function of the cell wall was disrupted by the RTMBE, the bacteria soon died.

### 2.4. SDS-PAGE Electrophoresis of the Bacterial Protein

**Figure 4:**
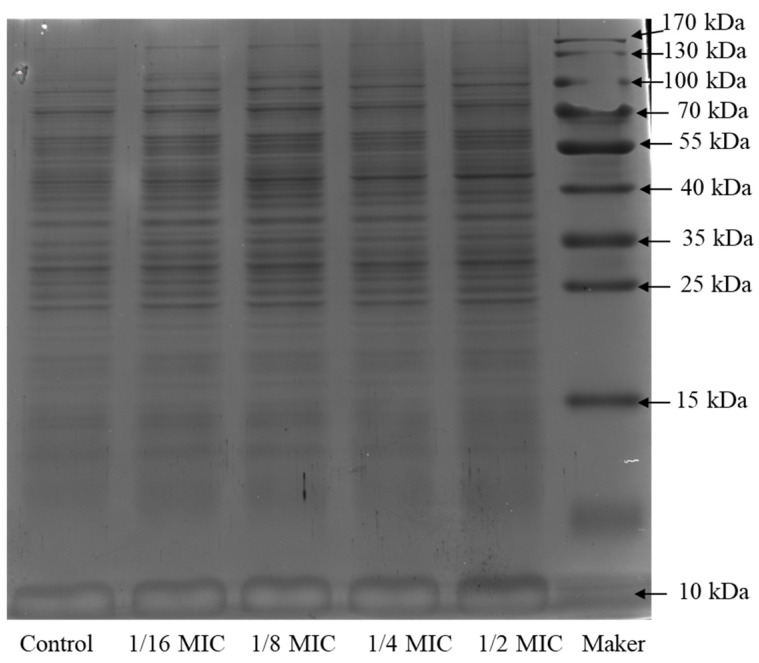
SDS-PAGE patterns of the total proteins for *Pcc*S1 treated by RTMBE.

It is shown in Figure 4 that the protein profiles of bacteria treated with RTMBE were similar with the untreated one. In the protein electrophoresis bands, the protein bands between 55 and 65 kDa became shallow and fainter when treated by 1/8 × MIC and 1/16 × MIC extract and even disappeared at the concentration of 1/2 × MIC and 1/4 × MIC. The protein band (40 kDa) was clear in the control group, whereas it became lighter when treated by 1/2 × MIC extract. It can be concluded that the RTMBE disturbed the protein synthesis and destroyed the membrane integrity of the bacteria, which resulted in the leakage of the total protein. However, some protein bands between 40 and 100 kDa were gradually augmented with the increased extract concentration, which could be ascribed to the degradation of a macromolecular protein or the chaperones and signal transduction protein produced by bacteria in the stressful environment [33]. This was different from the lighter protein bands [34]. According to the time–kill kinetics, 1/4 × MIC was determined to analyze the bacterial intracellular proteome profiling response to RTMBE treatment in this text.

### 2.5. Scanning Electron Microscopy (SEM) Analysis

**Figure 5:**
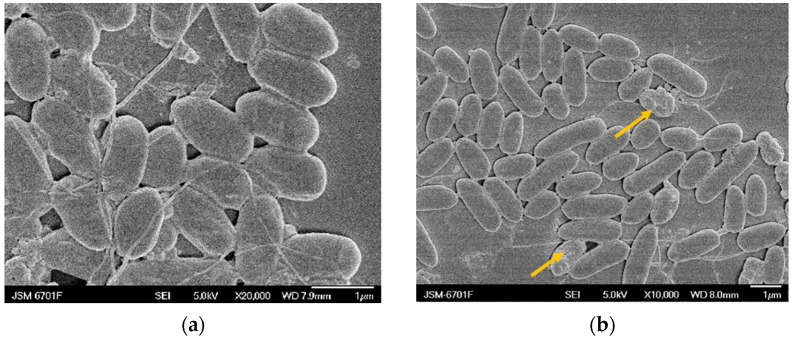

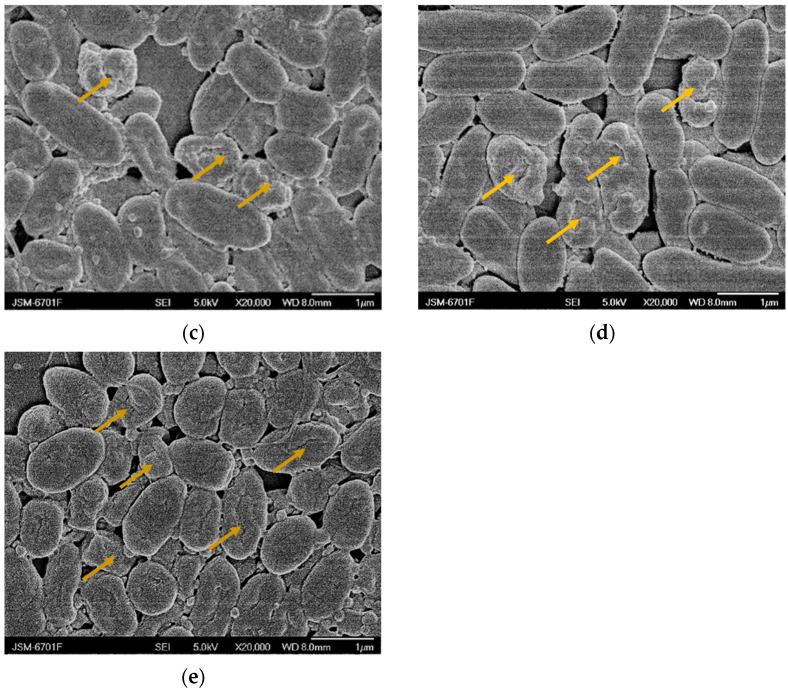
SEM images of *Pcc*S1 untreated (**a**) and treated by 1/8 × MIC (**b**), 1/4 × MIC (**c**), 1/2 × MIC (**d**) and 1 × MIC RTMBE (**e**). The yellow arrows represent sunken and irregular bacterial surfaces.

The SEM observations were carried out based on Reference [35], with slight modifications in the text. The *Pcc*S1 cells at the exponential phase were harvested and dissolved in PBS. Then, the RTMBE was added with different concentrations for 12 h. As shown in Figure 5a, the *Pcc*S1 cell was smooth, integral and typical short rod-shaped in the control group, whereas the cell membrane and cell wall integrity were gradually disrupted in the treatment. No visible holes or morphologic changes were observed after treatment with 1/8 × MIC RTMBE, except for a small number of cell surface wrinkles (Figure 5b); however, most of the cell membranes and cell walls were completely destroyed and became more shrunken and hollower in Figure 5e, resulting in cytoplasmic material leakage. In addition, the treated strain showed an obvious aggregate reaction, which may be ascribed to the quorum sensing produced by the bacteria in response to external stimulation [36]. The results supported that RTMBE has a severe impact on the permeability and integrity of cell walls [37]. It was speculated that the proteins associated with bacterial biofilms played an important role in understanding the antimicrobial mechanism.

### 2.6. Proteomic Analysis

**Figure 6:**
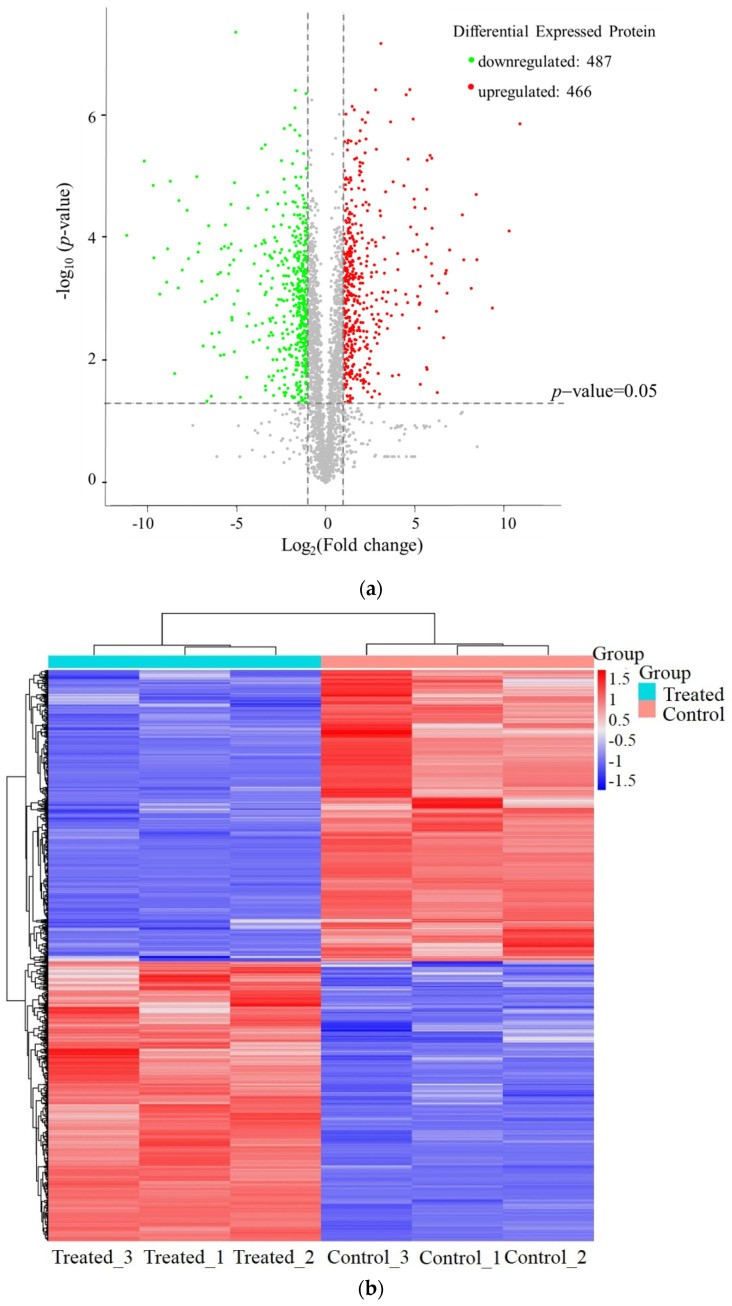
Label-free analysis reveals DEPs between the two groups. Volcano plot of the DEPs obtained from control and treatment samples, the red and green dots are considered to be differentially expressed proteins (DEPs) (**a**), The hierarchical clustering analysis (**b**).

In order to further investigate the effects of RTMBE on *Pcc*S1 at the molecular level, the label-free based LC-MS/MS quantitative proteomic approach was utilized. By using the MaxQuant program, a total of 3273 proteins derived from 28,372 unique peptides in the treated sample were identified, while there were 3258 protein groups detected in the untreated control sample. Among these proteins, the expression levels of 953 proteins were shown to be significantly different (FC > 2.0 or FC < 0.5, *p* < 0.05) between the treated and untreated bacteria, including 466 upregulated and 487 downregulated proteins. A hierarchical clustering analysis (Figure 6) was also applied to visualize the expression levels of all proteins in the groups, showing that the proteins were significantly changed using R studio software. To gain insight into the functional categories of the 953 different expressed proteins (DEPs), the identified proteins were further analyzed by GO annotation using the UniProt database, which is the main way to understand the gene functions.

**Figure 7:**
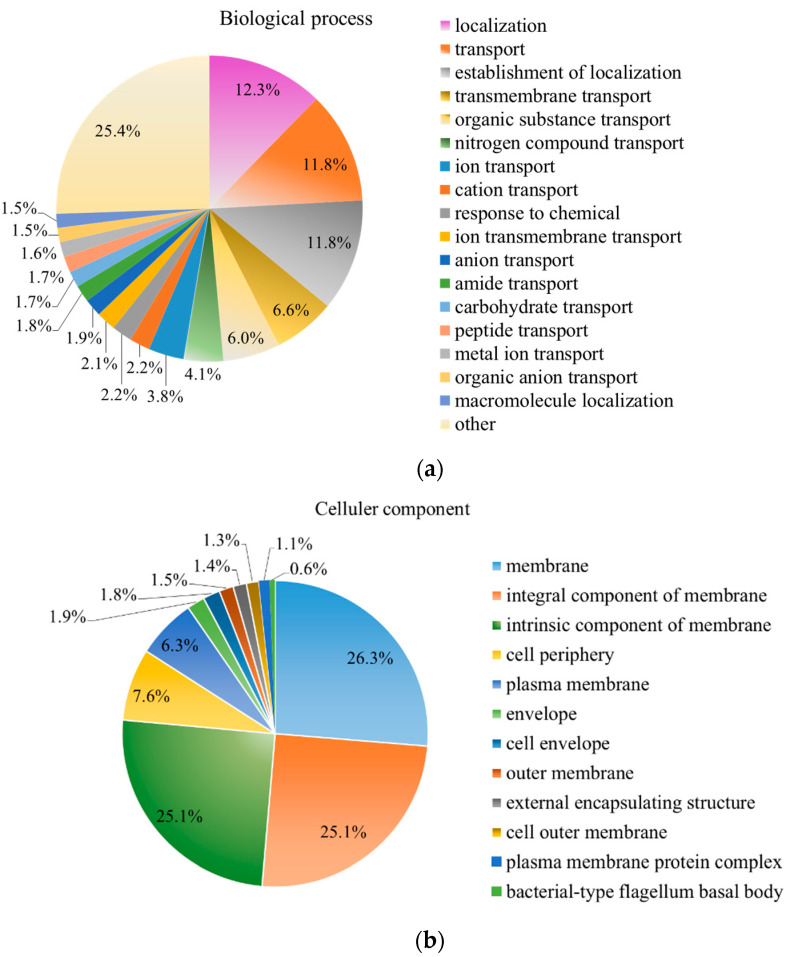

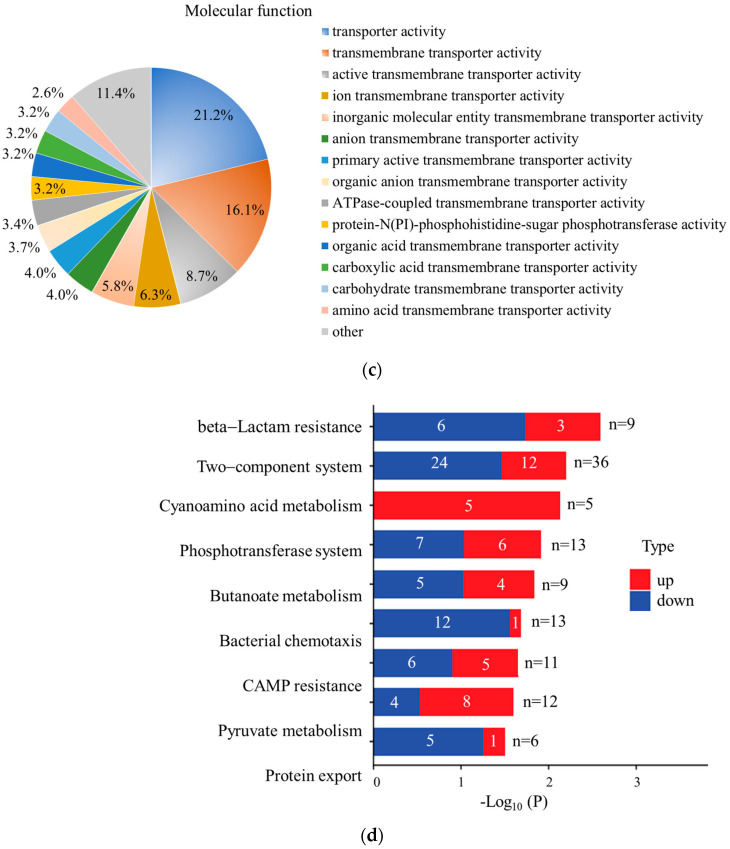
GO annotation of differentially expressed proteins after treatment with RTMBE, including a biological process (**a**), cellular component (**b**) and molecular function (**c**). KEGG pathway enrichment analysis (**d**). Experiments were performed in triplicate, and data are shown as the mean ± SD.

As shown in Figure 7, the majority of the enriched DEPs were mainly annotated into several cellular components (Figure 7a), including the membrane (26.3%), integral component of membrane (25.1%) and intrinsic component of membrane (25.1%). In the molecular functional category (Figure 7c), 21.2% of the proteins were related to transporter activity, followed by the transmembrane transporter activity (16.1%) and active transmembrane transporter activity (8.7%). The KEGG pathway analysis showed that the most notable pathway was beta-Lactam resistance (Figure 7d), a major determinant of this resistance in Gram-negative pathogens [38]. Previous studies showed that resistance to beta-lactam could be primarily ascribed to the presence of β-lactamase, the mutation of β-lactam targets and overexpression of efflux pumps [39]. In this case, type I secretion outer membrane protein TolC, penicillin-binding protein PBP1A, efflux pump membrane transporter and AmpG involved in beta-Lactam resistance were obviously downregulated. It was suggested that TolC could be a better target for the development of efflux pump inhibitors [40]. The downregulation of the TolC protein in this case indicated that *Rheum tanguticum* extract can be considered a natural antimicrobial agent, which may also increase the sensitivity to antibiotics.

The membrane-associated PBP1A protein is the main component of bacterial cell walls and can bind to the β-lactam antibiotic penicillin [41]. The protein PBP1A was downregulated after dealing with RTMBE, suggesting that its active ingredients may inhibit the formation or structural stabilization of cell walls. This was also consistent with the morphological tests of bacteria after RTMBE treatment by SEM and FTIR detection. The ampG encodes a transmembrane protein functioning as a specific permease to transport peptides, which are the signal molecules involved in ampC expression [42]. The membrane protein AmpG was significantly downregulated in this case, indicating that the permeability and stability of the cell membrane were repressed, and, correspondingly, the bacterial adhesion, invasion and resistance to complement sterilization and pathogenic processes were also affected. It was convinced that the outer membrane was seriously damaged and intracellular proteins were gradually released, which was consistent with the results of SDS-PAGE electrophoresis.

It was reported that the flagellar motility was considered as an accessory virulence determinant in plant bacterial pathogens [10,43]. Previous studies showed that the motility phenotypes of *Pcc*S1 were altered when some key genes, *mreB*, *flgK* and *hfq*, were knocked out; simultaneously, the virulence and plant cell wall-degrading enzyme activities were decreased [10]. From the downregulated flagellar motility-related protein in this text, such as flagellar hook protein FlgE, flagellar motor switch protein FliG, flagellar l-ring protein, flagellar motor protein MotA, flagellar basal-body rod protein FlgC and flagellar biosynthetic protein FliP, it was speculated that the RTMBE also played an essential antibacterial role by affecting the swimming motility of the cells. In addition, all of the proteins for pectate lyase were conversely upregulated after RTMBE treatment, which was similar to the previous work that genes of pectate lyase were positively plant-induced types during the bacteria inflection process [44]. This may ascribe to the bacterial general stress response, which plays a critical role in stress priming that increases bacterial fitness to threats from the environment [45]. In addition, the type I secretion outer membrane protein and virulence-related outer membrane protein were suppressed after dealing with RTMBE, indicated that the soft-rot pathogenic microbial decreased its ability to attack the host plants and compete with environmental bacteria [46,47].

### 2.7. Protein–Protein Interactions (PPI) Analysis

**Figure 8:**
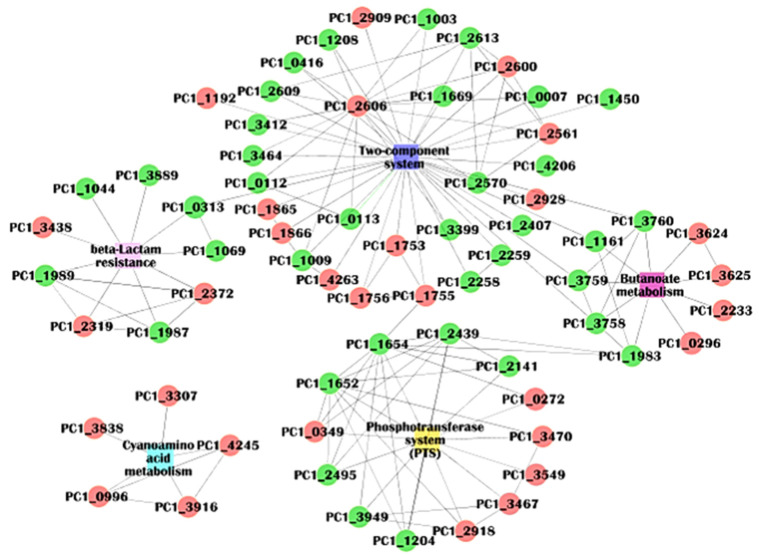
Protein–protein interactions analysis for DEPs and their pathways. Green node indicates a downregulated protein, and red means an upregulated one.

STRING is an online analysis tool used to analysis protein–protein interaction networks, which is a remarkable method for understanding the biological responses in health and disease [48]. As shown in Figure 8, the PPI network of the DEPs and their pathways contained 72 nodes and 145 edges, and the average number of neighbors was 4.028. The PPI enrichment *p*-value was 0.0025, which means more interactions among these proteins. Some proteins were related simultaneously to two pathways: butanoate metabolism and a two-component system, such as fumarate reductase subunit C (PC1_3760), 3-oxoacid CoA-transferase (PC1_1161), fumarate reductase iron-sulfur subunit (PC1_3759) and fumarate reductase flavoprotein subunit (PC1_3758). The response regulator receiver protein (PC1_2606) of the two-component system interacted with 17 proteins (15 proteins were downregulated), suggesting its essential role in the network interactions. The actual quantity of the edges was more than the expected quantity of the edges in the PPI of the DEPs, indicating that more interactions existed among these proteins, especially the suppressed ones.

## 3. Materials and Methods

### 3.1. Chemical Reagents, Bacterium and Growth Condition

*Rheum tanguticum* Maxim. ex Balf. was obtained from Gannan Tibetan Autonomous Prefecture more than 3000 m above the sea level (Gansu Province, China) and was identified by Professor X. Luo (Chemical Engineering, Northwest Minzu University, Lanzhou, China). Ammonium bicarbonate, dimethyl sulfoxide (DMSO), dithiothreitol (DTT), iodoacetamide (IAA) and sodium carbonate were purchased from Sigma-Aldrich (St. Louis, MO, USA). Urea and sodium dodecyl sulfate (SDS) were purchased from Bio-Rad (Hercules, CA, USA). Acetonitrile and water for nano-LC-MS/MS were purchased from J.T. Baker (Phillipsburg, NJ, USA). Trypsin was purchased from Promega (Madison, WI, USA). All other chemical reagents were analytical grade. The resistant *Pcc*S1 strain was obtained from the Key Laboratory of Integrated Management of Crop Diseases and Pests, Nanjing Agricultural University. The strain was activated in LB broth at 28 °C under shaking conditions.

### 3.2. Preparation of the Extract of the Natural Medicine

*Rheum tanguticum* was first crushed into small pieces and mixed, then soaked in 75% ethanol (1:10, *w*/*v*) and refluxed for 2 h. The filtrates were collected, and the residues were refluxed in 75% ethanol for 1.5 h. Two batches of filtrates were combined, filtered and evaporated with rotary evaporation; finally, the powder was obtained by using a vacuum freeze dryer. The RTMBE was adjusted to the corresponding concentration by adding DMSO (5% *v*/*v*). Afterwards, the solution was filtered through a 0.22-μm filter and stored in a seal at 4 °C.

### 3.3. Ultra-Performance Liquid Chromatography-Electrospray-Mass Spectrometry (UPLC-ESI-MS) Analysis

UPLC was performed by using a Thermo Scientific reversed phase C18 Column (100 mm × 2.1 mm, 3 μm). RTMBE was adjusted to 1 g/mL by methanol and filtered through a 0.22-μm filter. Chromatographic separations were performed at a column temperature of 30 °C, a flow rate of 20 μL/min and an injection volume of 0.5 μL. A linear gradient elution of methanol (A) and water with 1% formic acid (*v*/*v*) (B) was applied with the following program: 0–1 min, 5% A; 1–8 min, 5–20% A; 8–14 min, 20–20% A; 14–16 min, 20–25% A; 16–17 min, 25–27% A; 17–21 min, 27–32% A; 21–30 min, 32–32% A; 30–35 min, 32–35% A; 35–40 min, 35–40% A; 40–45 min, 40–50% A; 45–55 min, 50–90% A; 55–62 min, 90–95% A; 62–64 min, 95–95% A; 64–65 min, 95–5% A; 65–68 min, 5–5% A. The ultraviolet detection wavelength was set as 254 nm. Mass spectra were acquired with an ESI source in the range of *m*/*z* 100–1000. The optimized MS parameters were set as follows: spray voltage 3.78 Kv, spray current 16.03 µA, temperature 215.22 °C, sheath gas flow rate 19.99 arb, aux gas flow rate 4.99 arb, sweep gas flow rate 0.99 arb and capillary temperature 257.34 °C. Electrospray ionization was applied either in the negative mode to obtain better chromatograms. The software Elemental composition TM was used for data acquisition and processing.

### 3.4. The Minimum Inhibitory Concentration (MIC)

According to the method described in Reference [49], the MIC of RTMBE was determined by the twofold dilution method in a 96-well microplate. After cultivation in the log phase, the final concentration of bacteria suspension was adjusted to ~10^6^ CFU/mL. RTMBE with different concentrations were added in each hole. The bacteria cultured in LB without drugs was used as the positive control, while the liquid without bacteria was regarded as the negative control. Then, the color change of the mixture was assessed visually, and Microplate Reader determined the OD_600_ after incubating for 24 h at 28 °C. The lowest concentration inhibiting the growth of the strain was considered as the MIC [50]. All the tests were repeated three times.

### 3.5. Measurement of Antibacterial Curves

The strain *Pcc*S1 was cultivated in LB broth at 180 rpm and 28 °C; then, the cells were collected by centrifugation (8000× *g*, 10 min). When the OD_600_ value was 0.8, the bacterial suspensions were adjusted to 0.5 McFarland using fresh LB medium. Finally, the treated suspensions with RTMBE concentrations of 1 × MIC, 1/2 × MIC, 1/4 × MIC and 1/8 × MIC, respectively, were obtained. The OD_600_ of the supernatants was finally measured by an ultraviolet spectrophotometer to draw a bacteriostasis curve, which used the time as the abscissa and the OD_600_ value as the ordinate.

### 3.6. Measurement of Extracellular Protein Content

The *Pcc*S1 cells at exponential phase were treated for 2, 4, 6, 8, 10, 12, and 24 h using RTMBE in the same manner as antibacterial curves. After centrifugation, the supernatants were used to detect the extracellular protein content by Commassie Blue Staining Kit based on Bradford method (Nanjing Jiangcheng Bioengineering Institute, China). The supernatant turns blue if the protein sample is combined with the Coomassie Brilliant Blue G-250, the protein content can be calculated by measuring the absorbance:

$$\mathrm{The}\;\mathrm{protein}\;\mathrm{content}\;(g/L)\;=\frac{{\mathrm{OD}}_{595\mathrm{sample}}-{\mathrm{OD}}_{595\mathrm{blank}}}{{\mathrm{OD}}_{595\mathrm{standard}}-{\mathrm{OD}}_{595\mathrm{blank}}}\times \mathrm{Sc}\times \mathrm{Df}$$

where the Sc means the Standard concentration (g/L), the Df means the Dilution factor.

### 3.7. SDS-PAGE Analysis of Bacterial Intracellular Proteins

The strained cells in the exponential phase were treated with RTMBE at different concentrations for 8 h; then, the bacteria were collected by centrifugation at 8000× *g* for 10 min and washed three times with 0.1 M phosphate-buffered saline (PBS, pH 7.2). The protein was extracted from tissue samples using a SDT lysis buffer. The samples were boiled for 5 min and further ultrasonicated and boiled again for another 5 min. Undissolved cellular debris were removed by centrifugation at 16,000× *g* for 15 min. The supernatant was collected and quantified with a BCA protein assay kit (Bio-Rad, Hercules, CA, USA). Fifteen micrograms of bacterial suspension samples mixed with the loading buffer were boiled in tubes and then loaded on the gel. Electrophoresis was performed at 80 V through the stacking gel (5%) and at 120 V through the separation gel (12%). Coomassie brilliant blue R-250 was used to stain the gel, and the separated protein bands were obtained after being decolorized. The bacteria cultured in LB without treatment was used as the positive control. All the tests were repeated three times.

### 3.8. Fourier Transform Infrared Spectrophotometer (FTIR) Analysis

A FTIR analysis was applied to test the variations of particular chemical moieties of *Pcc*S1 affected by RTMBE. After the cultivation of these strains, respectively, with the 1 × MIC, 1/2 × MIC, 1/4 × MIC and 1/8 × MIC RTMBE concentrations, the cells were washed three times by 5 mL of PBS, centrifuged at 8000× *g* for 5 min and then dried at 28 °C. The presence of various functional groups in bacterial cells samples were identified by FTIR (Perkin Elmer Spectrophotometer 100, Waltham, MA, USA) using a KBr pellet in a range of 650–4000 cm^−1^ (64 scans and 1 cm^−1^ resolution). The obtained spectra were normalized, baseline-corrected and analyzed using SPECTRUM software.

### 3.9. Scanning Electron Microscopy (SEM)

The SEM analysis was performed based on the methods described in Reference [35] with slight modifications. The bacterial suspensions in the exponential phase were adjusted by using the PBS buffer, and then, RTMBE was added with the final concentration of 1 × MIC, 1/2 × MIC, 1/4 × MIC and 1/8 × MIC, respectively. The same volume of the buffer without drugs was considered as the control. After incubation for 8 h with shaking, the bacterial colonies were collected directly by washing and centrifugation (8000× *g* for 10 min). The bacterial cells were immobilized with a 2.5% glutaraldehyde solution at 4 °C for 6 h and then serially dehydrated by using gradient ethanol solutions (50–100%, *v*/*v*). Finally, the treated cells were coated with gold and observed using a Cold Field Emission Scanning Electron Microscope (JSM-6701F, 0.5 Kv–30 kV accelerating voltage, JEOL, Japan).

### 3.10. Protein Digestion and LC-MS/MS Analysis

Protein (200 μg for each sample) digestion was performed with the FASP method described by Reference [51]. In short, the detergent, DTT and IAA in UA buffer were added to block reduced cysteine. The protein suspension was digested with trypsin at a ratio of 50:1 overnight at 37 °C. The peptide was collected by centrifugation at 16,000× *g* for 15 min and desalted with C18 StageTip for further analysis. LC-MS/MS experiments were performed on a Q-Exactive Plus hybrid quadrupole-Orbitrap mass spectrometer coupled with Easy 1200 nLC (Thermo Fisher Scientific). Reverse-phase high-performance liquid chromatography (RP-HPLC) separation was performed with the EASY-nLC system at a 300-nL/min flow rate. The mobile phase was A (0.1% formic acid in water) and B (0.1% formic acid in 95% acetonitrile). MS data was acquired using a data-dependent top 20 method dynamically choosing the most abundant precursor ions from the survey scan (300–1800 *m*/*z*) for fragmentation. A lock mass of 445.120025 Da was used as an internal standard for mass calibration. The full MS scans were acquired at a resolution of 70,000 at *m*/*z* 200 and 17,500 at *m*/*z* 200 for the MS/MS scan. The MS data were analyzed using MaxQuant software and searching through the UniProt database. Label-free quantification was carried out using the intensity determination and normalization algorithm as previously described methods [52,53,54]. Only proteins with fold change (FC) > 2.0-fold (or FC < 0.5) and *p*-value < 0.05 were considered for significantly differential expressed proteins (DEPs).

### 3.11. Bioinformatics Analysis

Bioinformatics data were analyzed by Perseus software [55]. Hierarchical clustering analysis was performed with the Pheatmap package. UniProtKB/Swiss-Prot [56], Kyoto Encyclopedia of Genes and Genomes (KEGG) [57] and Gene Ontology (GO) [58] were used to extract and annotate the sequences. GO and KEGG enrichment analyses were carried out with Fisher’s exact test, and FDR correction for multiple testing was also performed. The protein–protein interaction (PPI) networks were constructed by using the STRING database and Cytoscape software [59].

### 3.12. Statistical Analysis

All data were expressed as the mean ± standard deviation (SD) of three replicates. One-way analysis of variance (ANOVA) and Tukey’s test were used to compare parametric data, and *p* < 0.05 was considered statistically significant in all experiments.

## 4. Conclusions

In this text, we reported for the first time that RTMBE has an evident inhibitory activity against the rifampicin- and streptomycin-resistant *Pcc* strain. The antibacterial mechanism of RTMBE inhibition may be related to the disturbance of the outer membrane proteins, which also play important roles in maintaining the integrity of the *Pcc*S1 cell membrane structure and the antibiotic resistance. It was indicated that RTMBE can be considered as good natural inhibitors, which can be used to circumvent β-lactamase-mediated resistance by combining with other pesticides. The results presented here show that RTMBE can decrease the virulence of the pathogenic bacteria and was considered as an alternative natural preserver for vegetables. In-depth further studying of the structure–activity relationship of the anthraquinone compound derivatives will help to discover more effective antibacterial lead compounds.

## Figures and Tables

**Table 1:** Characterization of the bioactive constituents of RTMB by UPLC-ESI-MS detection in the negative mode.

No.	RT (min)	Formula	Measured Mass [M-H]^−^ (*m*/*z*)	Theoretical Mass (*m*/*z*)	MS/MS	Identification	Reference
**1**	0.66	C_13_H_16_O_10_	331.0638	332.0638	125.0208,169.0153	Gallic acid-3-*O*-glucosidea	[19]
**2**	1.96	C_13_H_16_O_10_	331.0640	332.0671	125.0617, 169.0345	Gallic acid-4-*O*-glucosidea	[19].
**3**	7.05	C_15_H_14_O_6_	289.0718	290.0718	271.0652, 179.0033, 161.0523	catechin	[19]
**4**	7.50	C_30_H_26_O_12_	577.1264	578.1352	425.0423, 289.0742, 125.0241	Procyanidin B	[19,20]
**5**	17.03	C_23_H_26_O_11_	477.1453	478.1402	313.0147, 169.0135	Lindleyin	[19,21]
**6**	18.09	C_23_H_26_O_11_	477.1453	478.1402	313.0147, 169.0135	Isolindleyin	[19,21]
**7**	18.91	C_44_H_34_O_20_	881.1753	882.1571	729.1460, 559.1257, 541.0923 477.1452, 289.0909	procyanidin B-2-3,3′-di-*O*-gllate	[19,24]
**8**	22.44	C_19_H_22_O_19_	393.1192	394.4113	231.0256	6-Hydroxymusizin-8-*O*-b-d-glucopyranoside	[21,24]
**9**	25.25	C_21_H_18_O_11_	445.0702	446.0776	283.0281, 239.0978, 211.0514	Rhein-8-*O*-glucopyranoside	[19]
**10**	27.72	C_27_H_26_O_12_	541.1352	542.4321	313.0562, 227.0296, 169.0123	Resveratrol-4′-*O*-(2″-galloyl)-b-d-glucopyranoside	[20,23]
**11**	28.75	C_27_H_26_O_12_	541.1353	542.1352	313.0562, 227.0296, 169.0122	Resveratrol-4′-*O*-(6″-galloyl)-b-d-glucopyranoside	[20,23]
**12**	32.86	C_20_H_24_O_9_	407.1348	408.3990	313.0256, 245.0823, 230.0572	Torachrysone 8-*O*-glucoside	[21,22]
**13**	33.79	C_21_H_20_O_10_	431.0928	432.0984	269.0261, 240.9125, 211.0392 169.0077, 125.0299	Aloe-emodin-8-*O*-β-d-glucopyranoside	[19,23]
**14**	35.75	C_21_H_20_O_10_	431.0931	432.0984	269.0261, 240.0439, 225.0518	Emodin-1-*O*-β-d-glucoside	[19]
**15**	42.68	C_21_H_20_O_10_	431.0934	432.3780	269.0261, 239.0077, 211.0158	Aloe-emodin-3-(hydroxymethyl)-*O*-β-d-glucopyranoside	[19]
**16**	44.03	C_21_H_20_O_10_	431.0934	432.3780	269.0261, 253.0253, 225.0565 169.0077	Emodin-8-*O*-β- d-glucoside	[19,23]
**17**	44.95	C_32_H_32_O_13_	623.1873	624.1770	459.0034, 307.3884, 235.0591 169.0167, 125.0231	4-(4-Hydroxyphenyl)-2-butanone-4′-*O*-β-d-(6″-*O*-galloyl-2″-*O*-cinnamoyl)-glucopyranoside	[19]
**18**	45.55	C_32_H_32_O_13_	623.1763	624.1770	459.0032, 235.0581, 169.0167	4-(4-Hydroxyphenyl)-2-butanone-4 -*O*-β-d-(2″-*O*-galloyl-6″-*O*-p-coumaroyl)-glucopyranoside	[19]
**19**	46.82	C_22_H_22_O_10_	445.0703	446.1140	283.0281, 240.0381, 225.0498 212.0453	Rhein-8-*O*-β-d-glucopyranosid	[19,23]
**20**	47.81	C_30_H_30_O_15_	629.1561	630.2315	465.1231, 313.2586, 169.1358	4-(4′-Hydroxyphenyl)-2-butanone-4′-*O*-b-d-(2″-*O*-galloyl-6″-*O*-galloyl)glucopyranoside	[21]
**21**	48.27	C_32_H_32_O_12_	607.1840	608.1821	443.1002, 295.0456, 169.0345	4-(4′-Hydroxyphenyl)-2-butanone-4′-*O*-b-d-(2″-*O*-cinnamoyl-6″-*O*-galloyl)glucopyranoside	[19,21]
**22**	50.78	C_16_H_12_O_5_	283.0274	284.0612	240.0426	6-methyl-aloe emodin	[19]
**23**	53.14	C_15_H_10_O_5_	269.0427	270.0445	241.0371, 225.0597, 182.0562	emodin	[19]
**24**	54.17	C_15_H_10_O_4_	253.0505	254.0506	225.0534, 181.3284	Chrysophanol	[19]
**25**	55.49	C_16_H_12_O_5_	283.0608	284.0612	239.1295, 211.0185	Physcion	[19,23]

## Data Availability

All available data are contained within the article.

## Sample Availability

Samples of the compounds are not available from the authors.
